# Quality of Life and Mental Health in Mothers and Fathers Caring for Children and Adolescents with Rare Diseases Requiring Long-Term Mechanical Ventilation

**DOI:** 10.3390/ijerph17238975

**Published:** 2020-12-02

**Authors:** Johannes Boettcher, Jonas Denecke, Claus Barkmann, Silke Wiegand-Grefe

**Affiliations:** 1 Department of Child and Adolescent Psychiatry, Psychosomatics and Psychotherapy, University Medical Center Hamburg-Eppendorf, Martinistraße 52, 20251 Hamburg, Germany; barkmann@uke.de (C.B.); s.wiegand-grefe@uke.de (S.W.-G.); 2 Department of Pediatrics, University Medical Center Hamburg-Eppendorf, Martinistraße 52, 20251 Hamburg, Germany; j.denecke@uke.de

**Keywords:** parents, rare disease, gender differences, mechanical ventilation, mental health, quality of life

## Abstract

(1) Parents caring for children and adolescents with rare diseases fear the long-term progression of the child’s disease and the loss of their parental role. The aim of this study was to examine the quality of life, mental health and associated protective factors of mothers and fathers caring for children with rare diseases requiring mechanical long-term ventilation. (2) In a cross-sectional design, data on quality of life, mental health, coping mechanisms, social support and family functioning from *n* = 75 affected families were collected using standardized psychometric questionnaires. (3) Mothers compared to fathers were significantly more impaired in their quality of life and mental health. Protective factors significantly associated with the respective outcomes for mothers were coping mechanisms, social support and family functioning, whereas for fathers solely the latter was found to be significant. Multiple regression analyses showed that family functioning may be the most important predictor of quality of life and mental health. (4) The results support the need for family-oriented care in parents of children with rare diseases. To reach optimal efficiency, health care providers should not only screen parents for psychosocial impairment but also provide interventions that consider gender-specific differences in psychological health.

## 1. Introduction

Despite an increasing interest in patients with rare diseases over the last few years, parents as the caregivers of affected children have received little attention within the healthcare system and in healthcare research. Rare diseases are defined as diseases affecting less than one in 2000 people [1] and are characterized by a severe, chronic, often degenerative and life-shortening course as well as limited treatment options and a loss of independence in everyday life [2]. Therefore, rare diseases in childhood and adolescence have a tremendous impact on the psychosocial situation of all family members, since they demand a high level of care and disease management by the affected families [3]. Given the severity of most rare diseases and the number of people affected by them, their importance in health care is considerable [4]. In particular, mothers of children with rare diseases face long-term challenges and major sacrifices including social isolation and financial adversity as they mostly take the main caregiving role [5,6,7]. It is therefore not surprising that mothers and fathers of children with rare diseases experience different levels of stress that may lead to negative psychosocial outcomes in accordance with their gender [8].

Given the aforementioned factors, the Caregiving Process and Caregiver Burden Model provides a multidimensional framework that describes the caregiving burden of parents of children with chronic conditions [9]. This model incorporates previous frameworks that explored the mechanisms behind caregivers’ physical and psychological health [10,11,12], the latter including constructs such as quality of life (QoL) and mental health. The concept of QoL is described as “the individual’s perception of their position in life in the context of the culture and value systems in which they live, in relation to their goals, expectations, standards and concerns” [13]. The definition of mental health, on the other hand, comprises the “flexibility and ability to cope with adverse life events and function in social roles” [14]. According to the Caregiving Process and Caregiver Burden Model, psychological health is directly influenced by supportive factors, which include coping, social support and family functioning [9]. These factors have been confirmed primarily in caregiving mothers of children with cerebral palsy [15].

Evidence suggests that long-term caregiving for children and adolescents with rare diseases is associated with impairment in psychological health [3,16]. Among parents, those caring for rare disease-afflicted children and adolescents requiring mechanical long-term ventilation may be especially burdened [17,18]. The impairment of mental health in these parents is considerably high due to fears, insecurities and worries about the child’s life [19]. As a result of the enormous physical and psychological efforts by the parents, quite often mental health issues develop [17]. Not surprisingly, studies have found that parents caring for patients with mechanical long-term ventilation are at high risk of clinical depression [20,21]. Moreover, caregivers of technology-dependent children have been found to have a higher prevalence of depression and lowered QoL [20].

Resources and behaviors of parents that may play an important role in adjusting and adapting to the demands of caring for technology-dependent children include coping, social support and family functioning. In previous research, social support, was found to be a protective factor for psychosocial health outcomes in parents of technology-dependent children [20,21]. Moreover, family functioning has been significantly associated with symptoms of depression [22,23]. Coping mechanisms as important supportive factors for psychosocial outcomes in parents of technology-dependent children were only addressed briefly in previous research [21]. Although a gender-specific reaction regarding the perceived stressors may be expected, the experience of fathers has largely been neglected in the literature. As previous studies on caregivers with technology-dependent children put the emphasis on female rather than male caregivers, the specific psychosocial needs of fathers have not been explored sufficiently in this study population [17,20,21].

While it is obvious that caring for a child with a rare disease is a major challenge for affected parents, there is little quantitative research on this subject. In order to improve psychosocial support for parents caring for children and adolescents suffering from rare diseases that require mechanical long-term ventilation, it is essential to better understand various aspects and influencing factors of QoL and mental health. Since research remains biased towards focusing on mothers as caregivers of rare disease-afflicted patients, a focus on gender comparison between parents was set [8]. Moreover, in contrast to previous research, this study examined parents of children with a heterogeneous group of rare diseases embedded in a theoretical framework, using validated instruments. The following research questions were addressed:

1. Are there differences in the distribution of QoL and mental health between mothers and fathers caring for children and adolescents with rare diseases requiring mechanical ventilation?
2. Is there a significant association between coping and supportive factors and the psychosocial outcomes of QoL and mental health of affected parents?

In this regard, the following hypotheses were tested: (a) QoL and mental health are significantly lower in mothers than in fathers of rare disease-afflicted children; (b) there are significant differences between affected mothers and fathers regarding their coping mechanisms, social support and family functioning; and (c) general coping, social support and family functioning are significant predictors for QoL and mental health in parents of rare disease-afflicted children.

## 2. Materials and Methods

### 2.1. Study Design

The presented data are part of the CHROKODIL project, which investigated the psychosocial needs of families caring for a child or adolescent suffering from a severe chronic disease. In the case of the project presented here, the parents of children and adolescents with rare diseases requiring mechanical ventilation were investigated cross-sectionally by means of standardized psychometric questionnaires. The study represents an extension of the study conducted by Johannsen et al. (2020), which investigated psychosocial needs of the families of children with neuromuscular diseases [24]. The study was funded by the Werner Otto Foundation and received ethical approval by the Medical Chamber Hamburg (PV 4361).

### 2.2. Variables and Instruments

Quality of life: The Ulm Quality of Life Inventory for Parents (ULQIE) [25] assesses perceived QoL in parents of chronically ill children. The instrument consists of 29 items, which are answered on a five-point rating scale. Five respective subscales measure physical and daily functioning, satisfaction with family support, emotional strain due to the child’s illness, self-development and well-being. The total scale illustrating overall QoL is calculated by averaging all ratings, with higher scores indicating greater QoL. The ULQIE total score ranges from 0 to 4. The ULQIE has shown high internal consistency and high retest reliability in a sample of German parents of children with chronic diseases [25].

Mental health: The Brief Symptom Inventory (BSI) [26] is an internationally recognized screening method used to obtain initial information on mental health. The instrument consists of 53 items, which are answered on a five-point rating scale. Nine respective subscales measure somatization, obsession–compulsion, interpersonal sensitivity, depression, anxiety, hostility, phobic anxiety, paranoid ideation and psychoticism. Additionally, the Global Severity Index (GSI) is used to provide a composite score of overall distress by summing up the mean values of the nine symptom subscales. The GSI ranges from 0 to 36. Higher BSI scores indicate greater mental health. Sum scores can be converted into T-scores according to the normative population of the test manual. T-scores greater than or equal to 63 of the GSI or two or more subscales are defined as clinically significant [27]. The psychometric properties of the German version of the BSI were found to be good [28].

Coping: The Coping Health Inventory for Parents (CHIP) [28] is a 45-item checklist assessing parents’ self-reported coping patterns toward their child’s disease. It comprises the subscales (1) maintaining family integration, cooperation and an optimistic definition of the situation; (2) maintaining social support, self-esteem and psychological stability; and (3) understanding the health care situation through communication with other parents and consultation with medical staff. Items are rated on a four-point rating scale. Additionally, a total CHIP score is used by the sum of points for all items. The total CHIP score can range from 0 to 135, with higher scores representing greater use of the respective coping pattern. The German version of the CHIP has shown acceptable internal consistency and retest reliability [29].

Social support: The Oslo-Social Support Scale (OSSS-3) [30] is a brief checklist measuring the level of social support. It consists of three items asking about the number of people one can rely on regarding personal problems, the evaluation of third parties’ interest in oneself and the possibility of getting practical support from neighbors and friends. The higher the sum score, the stronger the social support, with scores ranging between 3 and 14. The German version of the OSSS-3 has shown good psychometric properties [31].

Family functioning: The German version of the Family Assessment Measure (FAM) [32] was used to describe the functioning of the family as a whole. It consists of 40 items, which are answered on a four-point rating scale. In addition to the seven following subscales: task accomplishment, role performance, communication, emotionality, affective involvement, control and values/norms, the FAM contains the two following control scales: social desirability and defense. In this study, the total FAM score of the general scale was solely used to capture the resources and problems of the families by summing up all values; the score ranged from 0 to 120. Higher scores indicate worse family functioning. The FAM has shown acceptable psychometric properties [32].

Socio-demographic and clinical variables: Parents completed a study-specific questionnaire about their gender, age, socioeconomic status, marital status and family structure. Clinical variables included classification of pediatric diseases that may require ventilation according to the guidelines of the German Society of Pneumology and Mechanical Ventilation [33], as well as types and duration of ventilation.

### 2.3. Sample

The eligibility criteria were as follows: Families were included in the study if they had at least one child under 21 years of age with a diagnosed severe disease, currently requiring mechanical ventilation or potentially requiring mechanical ventilation in the progression of the disease. Since data were also collected from the affected children, severe physical, mental or cognitive impairments, making participation impossible or unreasonable, were set as exclusion criteria. Written consent was obtained from all participants before enrollment. All participants were allowed to withdraw from the study at any given time.

Within the CHROKODIL project, 211 families with children or adolescents with severe chronic diseases either currently requiring ventilation or potentially prospectively requiring ventilation were identified between 2014 and 2015 from the long-term mechanical ventilation center “Lufthafen” of the Altonaer Children’s Hospital and the clinic for “Neuropediatrics” of the University Medical Center Hamburg–Eppendorf. Families were excluded due to lack of consent to participate (*n* = 65), lack of German language skills (*n* = 2), lack of contact data (*n* = 2) or due to the child’s age being over 21 (*n* = 9). Questionnaires were handed out to 134 families. The response rate was 57%. Written consent from 77 families was obtained. Additionally, two families had to be excluded due to the fact that either only the grandparents’ or only the ill child’s data were available. Finally, a total of 75 families with 110 parents including 72 mothers and 38 fathers participated in the study. Data for both parents were available for 35 families. Data for only 1parent were available for 37 mothers and 3 fathers. All children of the affected families were afflicted with a disease that met the definition of a rare disease according to the European Commission [1].

### 2.4. Statistics

Descriptive statistics of all variables and subscales were calculated. Differences between mothers and fathers were investigated using the *t*-test for dependent samples. To give an indication of the size of the effects, Cohen’s *d* was calculated. To investigate the bivariate associations between predictor and outcome variables, Pearson correlations were used. In order to define predictors of QoL and mental health, multiple linear regression models were conducted. Statistical significance was set at *p* < 0.05 (two-tailed). To address a possible bias of the partially overlapping sample, multiple imputation using the Markov Chain Monte Carlo (MCMC) approach was used. All analyses were conducted using SPSS Statistics 26 (IBM Corp., Armonk, NY, USA) and GraphPad Prism version 8 (GraphPad Software, San Diego, CA, USA).

## 3. Results

The main characteristics of the parents are shown in Table 1. The underlying classifications of pediatric diseases were central respiratory disorders (8.0%), restrictive ventilatory disorders (80.0%) and obstructive ventilatory disorders (12.0%). The age of the parents of the rare disease-afflicted children was significantly lower in mothers (M = 40.1, SD = 7.38) than in fathers (M = 43.1, SD = 7.38), *t* = −1.99, *p* = 0.049. The children’s ages ranged between 6 and 21 years (M = 9.5, SD = 5.48). The technology dependence of the children ranged between 0 and 24 h per day (M = 12.5, SD = 6.61).

### 3.1. Differences between Mothers and Fathers in Quality of Life

Table 2 shows the distribution of QoL according to the ULQIE scales for both mothers and fathers. Additionally, gender differences are given. On nearly all subscales, scores were lower for mothers than fathers. On the well-being and total score subscales, the scores of mothers were significantly lower compared to fathers, with effect sizes ranging from small to medium. This means that an average mother has a QoL that is half a standard deviation unit lower than that of an average father.

### 3.2. Mental Health

Table 3 shows the distribution and sex difference of mental health according to the BSI between mothers and fathers. Mothers showed higher scores in all subscales, with the interpersonal sensitivity, anxiety, hostility and global severity index subscales reaching significance. Respectively, effect sizes ranged from small to medium.

**Figure 1:**
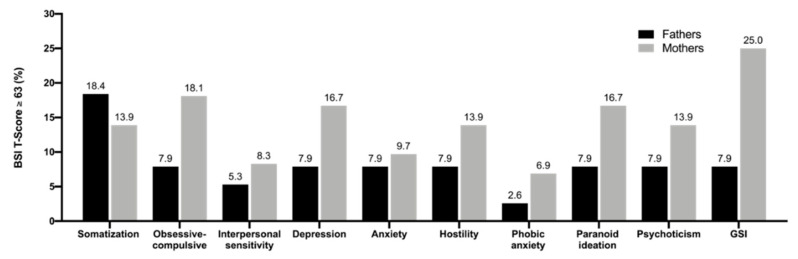
Percentage of mothers and fathers with clinically significant Brief Symptom Inventory (BSI) scores.

Figure 1 shows the proportion of BSI scores in mothers and fathers that are in the clinical range of mental health for each subscale and the GSI. In order to determine this proportion, the above-mentioned cut-off was used. Clinically significant cases were found in all domains for both mothers and fathers. In this regard, 31.2% of mothers and 13.2% of fathers scored to the point of clinical concern.

### 3.3. Differences between Mothers and Fathers in Coping and Supportive Factors

Table 4 shows the distribution and gender-specific comparison of coping and supportive factors in mothers and fathers. Significant differences in the following coping mechanisms: maintaining family integration (CHIP-FAM), understanding health care situation (CHIP-MED) and coping sum score (CHIP-Total), were found between mothers and fathers, with a small effect size. No other difference reached significance.

### 3.4. Predictors of QoL and Mental Health

Table 5 shows the Pearson correlation coefficients and levels of significance of possible associations between predictor and outcome parameters. For the mothers almost all predictors were significantly correlated with QoL and mental health, with the exception of the coping mechanism CHIP-FAM. In contrast, for the fathers the only significant correlations were found between QoL and family functioning as well as between mental health and family functioning.

Table 6 shows the multiple regression model with all predictors of QoL and mental health for both mothers and fathers. The analyses of QoL revealed that age of the child and family functioning were significant predictors of mothers’ QoL. The final model reached significance (*R*^2^ = 0.364, *p* < 0.001). Among fathers, the predictor family function was significantly associated with QoL. Again, the model could be confirmed (*R*^2^ = 0.180, *p* = 0.039). The multiple regression models with affiliated predictors of mental health are shown separately for mothers and fathers. Social support was a significant predictor for mothers’ mental health, with the model reaching significance (*R*^2^ = 0.362, *p* < 0.001). In contrast, significant predictors for mental health among fathers were ventilation and family function. The final model reached significance (*R*^2^ = 0.187, *p* = 0.015).

To investigate whether the different coping strategies as predictors have a different influence on the overall models of QoL and mental health, they were added individually to the multiple regression analyses. It was found that there was no major difference, whether using the individual total coping mechanisms or general coping mechanism as a predictor.

## 4. Discussion

Since rare diseases have a severe impact on patients and families, it is important that decisions regarding health services are based on data obtained from the affected families and children themselves [35]. The assessment of QoL and mental health of parents caring for children with rare diseases is therefore particularly valuable in customizing further support. Consequently, the present study explored QoL and mental health of parents caring for rare disease-afflicted children requiring mechanical long-term ventilation and supportive factors of the respective constructs.

Findings indicate that in families caring for children with rare diseases requiring long-term ventilation, mothers have significantly lower global QoL in comparison to their male counterparts. This is in line with previous research on parents of children with chronic diseases [36,37,38,39]. Although mothers had lower scores in most of the subscales compared to fathers, a significant difference could only be found for the well-being scale, which assesses perceived discomfort in the areas of physical activity and dejection. A possible explanation may be that mothers of children with a rare disease requiring long-term ventilation are the main caregivers of their children, leading to impairment in personal and professional life.

With regards to parental mental health, mothers reported significantly higher overall psychological distress in comparison to fathers, which is in line with previous findings on mental health of parents of chronically ill children [40,41]. Moreover, mothers reported higher psychological distress in all subscales. A more nuanced view of the mental health subscales shows that mothers compared to fathers showed significantly higher scores in the somatization, obsessive-compulsive, interpersonal sensitivity, depression, anxiety and hostility subscales. These findings correspond to the findings that close to a third (31.2%) of the affected mothers showed clinically relevant emotional distress, whereas only more than a tenth (13.2%) of the fathers showed this. The findings on the respective constructs demonstrate that the emotional burden of the child’s illness is more noticeable in mothers than in fathers. Therefore, these results on parental QoL and mental health confirm our first hypothesis.

Regarding protective factors, mothers in comparison to fathers showed significantly greater use of overall coping mechanisms. Moreover, the maintaining family integration and understanding the health care situation coping mechanisms were significantly more used by mothers than fathers. No other differences between affected mothers and fathers could be found regarding the remaining protective factors. These findings on the mothers’ greater use of coping mechanisms may be a consequence of the heightened burden due to the extensive caregiving required by the child’s disease. This goes along with a study that found the same gender differences in the use of coping strategies in parents of children with chronic renal failure [36]. Significant correlates of QoL and mental health in mothers were overall coping mechanisms, social support and family functioning, whereas in fathers only family functioning was associated with the respective outcomes. The second hypothesis on adaptive coping mechanisms can therefore solely be confirmed for the affected mothers. In particular the strong associations between the main outcomes QoL and mental health and the predictor family functioning in affected parents is in accordance with previous research, showing that better family functioning is associated with better parental mental health [21,22].

In the multiple regression analyses, family functioning was identified as a risk factor for mothers’ and fathers’ QoL, while also being a significant risk factor for mental health in mothers. This not only corresponds to the Caregiving Process and Caregiver Burden Model [9] but can also be found in a previous study on caregivers of technology-dependent children [20]. These findings suggest that family functioning may play a central role in the psychological health of affected caregivers. Therefore, healthcare providers working with families of children with rare diseases should support and care for the family as a whole in a family-based intervention approach. Besides family functioning, age of the child was found to be a significant predictor of QoL in mothers, with QoL increasing with age. In addition, the child’s current or prospective dependency on mechanical long-term ventilation was found to be a significant predictor of mental health in fathers, with higher psychological distress associated with the child’s dependency on mechanical long-term ventilation. Social support was found to be the only significant predictor of mental health in mothers. In previous studies, similar findings have been found. Social support was found to be the only significant predictor of mental health in mothers. In previous studies similar findings have been found, with mothers caring for ventilator-assisted children at home reporting greater social support, the lower the depression rate [21]. In summary, it seems that the key mechanism for psychological health outcomes in parents of children with rare diseases may be the family unit, whereas protective factors like social support and coping mechanisms may only play a secondary role. These findings are in concordance with a study that verified the Caregiving Process and Caregiver Burden Model in mostly female caregivers of children with cerebral palsy [15]. The third hypothesis can therefore only partly be confirmed in this study population.

These findings also highlight a difference in caregiver burden between mothers and fathers. This may be explained by the fact that the caregiving role in this study population was very traditional, with over 70% of mothers having predominantly taken over the primary caregiving of the afflicted child. This might explain the gender differences in the associated stressors of having a child with a rare disease.

### Study Limitations

There are some limitations to consider with this study. Even though the heterogeneity of rare diseases may be limited in this small sample, the only consistent characteristic being the children’s current or prospective dependency on mechanical long-term ventilation, the study sample does represent severe somatic diseases, all of which are associated with a particularly high need for care and a high level of disease management. Moreover, the study group represents a sample challenging to recruit. Patients were recruited at two institutions in northern Germany; thus, a transfer of results to countries with differing health care systems should only be done with caution. In light of these limitations, the results of this study should be considered preliminary. A replication of these results through international and longitudinal research studies seems desirable.

## 5. Conclusions

Our findings contribute to the existing literature by showing that there are significant differences in mothers and fathers of children with rare diseases requiring mechanical long-term ventilation regarding their QoL and mental health. Mothers seem to be more negatively impacted by their child’s disease than their male counterparts. Caring for a child with a rare disease can be seen as an ongoing life stressor, for which intrapersonal adaptation in particular seems to be essential. Psychosocial support services should therefore aim at strengthening the family situation, overcoming social isolation, strengthening intra-familial relationships and reinforcing coping strategies for the handling of the child’s disease. Finally, since the mothers’ evaluation suggests that mechanical ventilation does not have adverse effects on either their QoL or mental health, an early initiation of ventilatory assistance may be recommended.

## Figures and Tables

**Table 1:** Demographic data of study sample (*n* = 75 families).

Characteristics	*n*	%
Children’s gender		
Female	26	34.7
Male	49	65.3
Marital status		
Single Parent	8	10.5
Married	53	71.0
Divorced	14	18.4
Socioeconomic status of the families		
Low (*n*; %)	10	13.3
Moderate (*n*; %)	33	44.0
High (*n*; %)	32	42.7
Family structure		
One-child family	54	75.0
Two-child family	14	19.4
Three-child family	7	9.7
Child’s dysfunction		
Isolated pathology	6	7.9
Multiple disabilities without progression	23	30.3
Multiple disabilities with progression	4	5.3
Neuromuscular diseases	43	56.6
Ventilation		
No Mask	25	33.3
Mask	25	33.3
Tracheostoma	25	33.3

Note: socioeconomic status of the families computed according to Winkler and Stolzenberg (2009) [34].

**Table 2:** Comparison of quality of life (QoL) between mothers and fathers of children and adolescents with rare diseases requiring mechanical long-term ventilation.

	Mothers		Fathers		*p*	*d*
	M	SD	M	SD		
Physical and daily functioning	2.6	0.75	2.8	0.68	0.087	−0.27
Satisfaction with family	3.1	0.68	3.2	0.67	0.749	−0.05
Emotional stability	2.2	0.79	2.4	0.76	0.080	−0.25
Self-development	1.8	0.83	1.8	0.77	0.973	0.01
Well-being	2.6	0.66	3.1	0.63	0.001 ***	−0.58
Total score	2.5	0.54	2.7	0.52	0.014 *	−0.37

Note: Ulm Quality of Life Inventory for Parents (ULQIE) subscales. For means, standard deviations are given. Comparison between groups is assessed with two-sample *t*-test for dependent samples. * *p* ≤ 0.05, *** *p* ≤ 0.001, *d* = Cohen’s *d*.

**Table 3:** Comparison of mental health between mothers and fathers of children and adolescents with rare diseases currently or potentially prospectively requiring mechanical long-term ventilation.

	Mothers		Fathers		*p*	*d*
	M	SD	M	SD		
Somatization	2.5	3.54	1.4	2.31	0.093	0.340
Obsessive-compulsive	3.5	3.41	2.4	2.83	0.077	0.326
Interpersonal sensitivity	1.9	2.11	0.8	1.89	0.003 **	0.510
Depression	2.1	2.14	1.3	2.36	0.066	0.356
Anxiety	2.2	2.23	1.0	1.42	0.003 **	0.588
Hostility	2.2	2.07	1.3	1.78	0.020 *	0.456
Phobic anxiety	0.6	1.14	0.4	0.71	0.157	0.266
Paranoid ideation	2.3	2.74	1.6	2.38	0.169	0.263
Psychoticism	0.9	1.26	0.6	1.34	0.257	0.202
Global severity index	19.9	16.48	11.9	13.77	0.006 **	0.446

Note: Mean values and standard deviations for sum scores, with higher scores indicating higher psychological distress. *d =* Cohen’s *d.* Global Severity Index = sum of all items of the Brief Symptom Inventory. * *p* < 0.05, ** *p* < 0.01.

**Table 4:** Distribution of coping, social support and family functioning of parents of children and adolescents with rare diseases requiring mechanical long-term ventilation and comparison of mothers and fathers.

	Mothers		Fathers		*p*	*d*
	M	SD	M	SD		
CHIP-FAM	40.7	8.35	38.0	8.01	0.067	0.328
CHIP-SUP	38.6	6.87	37.1	7.31	0.180	0.218
CHIP-MED	15.7	4.29	14.1	4.05	0.033 *	0.378
CHIP-Total	95.0	17.67	89.2	17.58	0.056	0.329
OSSS	9.7	2.76	9.7	2.39	0.839	0.022
FAM	23.9	7.02	24.9	7.29	0.407	0.141

Note: For means, standard deviations are given. CHIP-FAM = maintaining family integration, CHIP-SUP = maintaining social support, CHIP-MED = understanding health care situation, OSSS = Oslo Social Support Scale. FAM = Family Assessment Measure. *d* = Cohen’s *d*. * *p* < 0.05.

**Table 5:** Pearson correlation between predictor and outcome parameters.

	Quality of Life		Mental Health	
	Mothers	Fathers	Mothers	Fathers
CHIP-FAM	0.12	−0.01	−0.12	−0.02
CHIP-SUP	0.34 **	0.08	−0.30 *	−0.17
CHIP-MED	0.25 *	0.07	−0.25 *	−0.16
CHIP-Total	0.25 *	0.05	−0.23 *	−0.12
OSSS	0.37 **	0.21	−0.47 **	−0.07
FAM	−0.46 **	−0.34 **	0.44 **	0.30 *

Note: CHIP-FAM = maintaining family integration, CHIP-SUP = maintaining social support, CHIP-MED = understanding health care situation, CHIP-Total = coping sum score, OSSS = Oslo Social Support Scale, FAM = Family Assessment Measure. * *p* < 0.05, ** *p* < 0.01.

**Table 6:** Predictors of quality of life and mental health in mothers and fathers.

	**Predictors of Quality of Life**							
	**Mothers**				**Fathers**			
	***b***		***SE***	***p***	***b***		***SE***	***p***
Intercept	1.840		0.574	0.001 **	3.274		0.780	0.001 ***
Age of child	0.024		0.011	0.030 *	0.001		0.015	0.937
Ventilation	0.013		0.122	0.914	−0.158		0.161	0.327
CHIP-Total	0.005		0.003	0.114	0.001		0.005	0.995
OSSS	0.045		0.027	0.102	0.038		0.035	0.278
FAM	−0.022		0.010	0.038 *	−0.033		0.013	0.009 **
	*F*	*df*	*p*	*R* ^2^_adj_	*F*	*df*	*p*	*R* ^2^_adj_
	8.12	5.69	0.001	0.319	3.09	5.69	0.039	0.121
	**Predictors of Mental Health**							
	**Mothers**				**Fathers**			
	***b***		***SE***	***p***	***b***		***SE***	***p***
Intercept	40.589		15.186	0.008 **	0.595		13.819	0.966
Age of child	−0.388		0.308	0.208	−0.222		0.218	0.307
Ventilation	2.008		3.415	0.557	6.138		2.502	0.014 *
CHIP-Total	−0.132		0.094	0.163	−0.074		0.090	0.411
OSSS	−1.949		0.690	0.005 **	0.032		0.591	0.956
FAM	0.541		0.277	0.051	0.632		0.225	0.005 **
	*F*	*df*	*p*	*R* ^2^_adj_	*F*	*df*	*p*	*R* ^2^_adj_
	7.97	5.69	0.001	0.316	3.19	5.69	0.015	0.128

Note: ULQIE total score and BSI Global Severity Index. Ventilation: Yes = 1 and No = 0. CHIP-Total = coping sum score, OSSS = Oslo Social Support Scale, FAM = Family Assessment Measure. * p < 0.05, ** p < 0.01, *** p < 0.001.

## References

[1] European Commission Rare Diseases. European Commission, European Union. http://europa.eu.int/comm/health/ph_threats/non_com/rare_diseases_en.htm.

[2] EURORDIS Rare Diseases: Understanding this Public Health Priority. http://beta.eurordis.org/IMG/pdf/princeps_document-EN.pdf.

[3] Pelentsov L.J., Fielder A.L., Laws T.A., Esterman A.J. (2016). The supportive care needs of parents with a child with a rare disease: Results of an online survey. BMC Fam. Pract..

[4] Nguengang Wakap S., Lambert D.M., Olry A., Rodwell C., Gueydan C., Lanneau V., Murphy D., Le Cam Y., Rath A. (2020). Estimating cumulative point prevalence of rare diseases: Analysis of the Orphanet database. Eur. J. Hum. Genet..

[5] Anderson M., Elliott E., Zurynski Y. (2013). Australian families living with rare disease: Experiences of diagnosis, health services use and needs for psychosocial support. Orphanet J. Rare Dis..

[6] Zurynski Y., Deverell M., Dalkeith T., Johnson S., Christodoulou J., Leonard H., Elliott E. (2017). Australian children living with rare diseases: Experiences of diagnosis and perceived consequences of diagnostic delays. Orphanet J. Rare Dis..

[7] Swallow V., Macfadyen A., Santacroce S.J., Lambert H. (2012). Fathers’ contributions to the management of their child’s long-term medical condition: A narrative review of the literature. Health Expect..

[8] Pelentsov L.J., Laws T.A., Esterman A.J. (2015). The supportive care needs of parents caring for a child with a rare disease: A scoping review. Disabil. Health J..

[9] Raina P., O’Donnell M., Schwellnus H., Rosenbaum P., King G., Brehaut J., Russell D., Swinton M., King S., Wong M. (2004). Caregiving process and caregiver burden: Conceptual models to guide research and practice. BMC Pediatrics.

[10] Pearlin L.I., Mullan J.T., Semple S.J., Skaff M.M. (1990). Caregiving and the Stress Process: An Overview of Concepts and Their Measures 1. Gerontologist.

[11] King G., King S., Rosenbaum P., Goffin R. (1999). Family-Centered Caregiving and Well-Beingof Parents of Children With Disabilities:Linking Process With Outcome. J. Pediatrics Psychol..

[12] Wallander J.L., Varni J.W., Babani L., Dehaan C.B., Wilcox K.T., Banis H.T. (1989). The Social Environment and the Adaptation of Mothers of Physically Handicapped Children 1. J. Pediatrics Psychol..

[13] (1995). The World Health Organization Quality of Life Group The World Health Organization quality of life assessment (WHOQOL): Position paper from the World Health Organization. Soc. Sci. Med..

[14] Galderisi S., Heinz A., Kastrup M., Beezhold J., Sartorius N. (2015). Toward a new definition of mental health. World Psychiatry.

[15] Raina P., Rosenbaum P., Brehaut J., Walter S.D., Russell D., Swinton M., Zhu B., Wood E. (2005). The Health and Well-Being of Caregivers of Children With Cerebral Palsy. Pediatrics.

[16] Waldboth V., Patch C., Mahrer-Imhof R., Metcalfe A. (2016). Living a normal life in an extraordinary way: A systematic review investigating experiences of families of young people’s transition into adulthood when affected by a genetic and chronic childhood condition. Int. J. Nurs. Stud..

[17] Mesman G.R., Kuo D.Z., Carroll J.L., Ward W.L. (2013). The Impact of Technology Dependence on Children and Their Families. J. Pediatrics Health Care.

[18] Carnevale F.A., Alexander E., Davis M., Rennick J., Troini R. (2006). Daily living with distress and enrichment: The moral experience of families with ventilator-assisted children at home. Pediatrics.

[19] Lee J., Lynn F. (2017). Mental health and well-being of parents caring for a ventilator-dependent child. Nurs. Child. Young People.

[20] Chan Y.H., Lim C.Z.-R., Bautista D., Malhotra R., Østbye T. (2019). The Health and Well-Being of Caregivers of Technologically Dependent Children. Glob. Pediatr. Health.

[21] Kuster P.A., Badr L.K. (2006). Mental health of mothers caring for ventilator-assisted children at home. Issues Ment. Health Nurs..

[22] Toly V.B., Bolton F.P., Musil C.M., Carl J.C. (2012). Families With Children Who Are Technology Dependent: Normalization and Family Functioning. West. J. Nurs. Res..

[23] Toly V.B., Musil C.M., Carl J.C. (2012). A longitudinal study of families with technology-dependent children. Res. Nurs. Health.

[24] Johannsen J., Fuhrmann L., Grolle B., Morgenstern L., Wiegand-Grefe S., Denecke J. (2020). The impact of long-term ventilator-use on health-related quality of life and the mental health of children with neuromuscular diseases and their families: Need for a revised perspective?. Health Qual. Life Outcomes.

[25] Goldbeck L., Storck M. (2002). Das Ulmer Lebensqualitäts-Inventar für Eltern chronisch kranker Kinder (ULQIE). Z. Klin. Psychol. Psychother..

[26] Derogatis L.R., Melisaratos N. (1983). The Brief Symptom Inventory: An Introductory Report. Psychol. Med..

[27] Franke H. (2000). Brief Symptom Inventory von L.R. Derogatis (Kurzform der SCL-90-R). Deutsche Version. Manual.

[28] Geisheim C., Hahlweg K., Fiegenbaum W., Frank M. (2002). Das Brief Symptom Inventory (BSI) als Instrument zur Qualitätssicherung in der Psychotherapie. Diagnostica.

[29] McCubbin H.I., McCubbin M.A., Cauble E., Goldbeck L. (2001). Fragebogen zur elterlichen krankheitsbewältigung: Coping health inventory for parents (CHIP)-deutsche version. Kindh. Entwickl..

[30] Meltzer H., Nosikov A., Gudex C. (2003). Development of a Common Instrument for Mental health.

[31] Kocalevent R.D., Berg L., Beutel M.E., Hinz A., Zenger M., Härter M., Nater U., Brähler E. (2018). Social support in the general population: Standardization of the Oslo social support scale (OSSS-3). BMC Psychol..

[32] Cierpka M., Frevert G. (1994). Die Familienbögen: Ein Inventar zur Einschätzung von Familienfunktionen.

[33] Windisch W., Dreher M., Geiseler J., Siemon K., Brambring J., Dellweg D., Grolle B., Hirschfeld S., Köhnlein T., Mellies U. (2017). Guidelines for non-invasive and invasive home mechanical ventilation for treatment of chronic respiratory failure-update 2017. Pneumologie.

[34] Winkler J., Stolzenberg H. (2009). Adjustierung des Sozialen-Schicht-Index für die Anwendung im Kinder- und Jugendgesundheitssurvey (KiGGS).

[35] Baumbusch J., Mayer S., Sloan-Yip I. (2019). Alone in a Crowd? Parents of Children with Rare Diseases’ Experiences of Navigating the Healthcare System. J. Genet. Couns..

[36] Wiedebusch S., Konrad M., Foppe H., Reichwald-Klugger E., Schaefer F., Schreiber V., Muthny F.A. (2010). Health-related quality of life, psychosocial strains, and coping in parents of children with chronic renal failure. Pediatr. Nephrol..

[37] Rensen N., Steur L.M.H., Schepers S.A., Merks J.H.M., Moll A.C., Kaspers G.J.L., Van Litsenburg R.R.L., Grootenhuis M.A. (2020). Determinants of health-related quality of life proxy rating disagreement between caregivers of children with cancer. Qual. Life Res..

[38] Witvliet M., Sleeboom C., De Jong J., Van Dijk A., Zwaveling S., Van Der Steeg A. (2014). Anxiety and quality of life of parents with children diagnosed with an anorectal malformation or hirschsprung disease. Eur. J. Pediatrics Surg..

[39] Jones B.L., Pelletier W., Decker C., Barczyk A., Dungan S.S. (2010). Fathers of children with cancer: A descriptive synthesis of the literature. Soc. Work Health Care.

[40] van Oers H.A., Haverman L., Limperg P.F., van Dijk-Lokkart E.M., Maurice-Stam H., Grootenhuis M.A. (2014). Anxiety and depression in mothers and fathers of a chronically ill child. Matern. Child Health J..

[41] Malm-Buatsi E., Aston C.E., Ryan J., Tao Y., Palmer B.W., Kropp B.P., Klein J., Wisniewski A.B., Frimberger D. (2015). Mental health and parenting characteristics of caregivers of children with spina bifida. J. Pediatrics Urol..

